# Case Report: Novel Biallelic Null Variants of SMPD4 Confirm Its Involvement in Neurodevelopmental Disorder With Microcephaly, Arthrogryposis, and Structural Brain Anomalies

**DOI:** 10.3389/fgene.2022.872264

**Published:** 2022-05-16

**Authors:** Weigang Ji, Xiangtian Kong, Honggang Yin, Jian Xu, Xueqian Wang

**Affiliations:** ^1^ Department of Pediatrics, Affiliated Matern & Child Care Hospital of Nantong University, Nantong, China; ^2^ Department of Prenatal Screening and Diagnosis Center, Affiliated Matern & Child Care Hospital of Nantong University, Nantong, China; ^3^ Department of Radiology, Affiliated Matern & Child Care Hospital of Nantong University, Nantong, China; ^4^ Department of Ultrasound, Affiliated Matern & Child Care Hospital of Nantong University, Nantong, China; ^5^ Nantong Institute of Genetics and Reproductive Medicine, Affiliated Matern & Child Care Hospital of Nantong University, Nantong, China

**Keywords:** *SMPD4*, neurodevelopmental disorder (NDD), null variants, microcephaly, structural brain anomalies, arthrogryposis, early death

## Abstract

The *SMPD4* gene encodes sphingomyelin phosphodiesterase 4, which preferentially hydrolyzes sphingomyelin over other phospholipids. The biallelic loss-of-function variants of *SMPD4* have been identified in a group of children with neurodevelopmental disorder with microcephaly, arthrogryposis, and structural brain anomalies (NEDMABA). Here, we report a girl of Chinese ancestry with intrauterine growth restriction, microcephaly, postnatal developmental delay, arthrogryposis, hypertonicity, seizure, and hypomyelination on brain magnetic resonance imaging; biallelic null variants (c.1347C > G [p.Tyr449*]; Chr2 [GRCh37]: g.130877574_131221737del [whole-gene deletion]) were detected by whole-exome sequencing. Our case is the first report of NEDMABA of Chinese ancestry, confirming the involvement of *SMPD4* in NEDMABA and expanding the mutation spectrum of this syndrome.

## Introduction

Neurodevelopmental disorders are a group of highly heterogenous conditions characterized by an inability to reach cognitive, emotional, and motor developmental milestones. Neurodevelopmental disorders have a complex pathophysiology and an etiology that may involve genetic and environmental factors such as genetic syndromes, metabolic abnormalities, immunologic disorders, infection, physical trauma, and exposure to toxic agents. Many neurodevelopmental disorders accompanied by structural abnormalities have a chromosomal or monogenic etiology. Recently, neurodevelopmental disorder with microcephaly, arthrogryposis, and structural brain anomalies (NEDMABA) was described as an autosomal recessive disorder (Mendelian Inheritance in Man [MIM]: 618622) caused by homozygous or compound heterozygous mutations in the sphingomyelin phosphodiesterase 4 gene (*SMPD4*; MIM: 610457) on chromosome 2 (Magini et al., 2019). The majority of individuals with NEDMABA present with intrauterine growth restriction (IUGR), congenital microcephaly, neonatal respiratory distress, and arthrogryposis of the hands and feet. Magnetic resonance imaging (MRI) has revealed a simplified gyral pattern of the cerebral cortex, delayed myelination, thin corpus callosum, and hypoplasia of the brainstem and cerebellum (Magini et al., 2019; Monies et al., 2019; Ravenscroft et al., 2020).

Here, we report two compound heterozygous null variants of *SPMD4* in a child of a Chinese family presenting with IUGR, microcephaly, postnatal developmental delay, hypomyelination, arthrogryposis, hypertonicity, and seizures. As the clinical manifestations were identical to NEDMABA, our findings provide additional evidence for the critical role of *SMPD4* in this syndrome.

## Case Presentation

**FIGURE 1:**
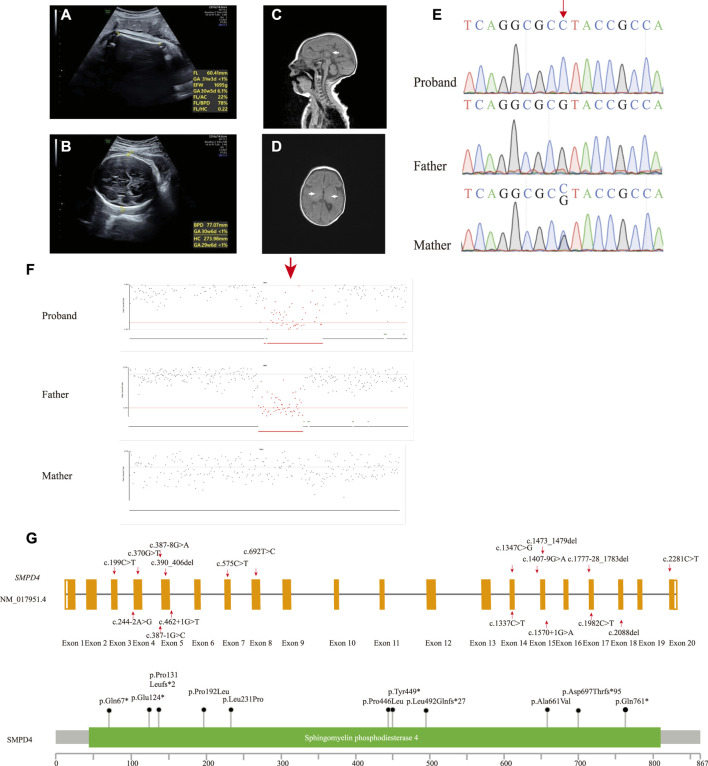
**(A,B)** Prenatal ultrasound image of fetus at 34 + 3 weeks showing IUGR (A) and microcephaly **(B)**. MR image of the 2-month-old patient. **(C)** T1-weighted image (T1WI) of median sagittal section showing thin corpus callosum, splenium, and unclear body (white arrow). **(D)** T1WI axial section absent of high signal of bilateral posterior limbs of the internal capsule showing hypomyelination (white arrow). **(E)** Sanger sequencing results of the proband and her parents. The single-nucleotide substitution is indicated by the red arrow. **(F)** Deletion in the proband was inherited from the father (red arrow). **(G)** Schematic presentation of linear SMPD4 transcript (NM_017951.4) (up) and protein (down) with all variants reported.

A girl was the first child of a nonconsanguineous couple of Chinese ancestry. Her family history was unremarkable. She was naturally conceived when her mother was 22 years old. At the first trimester screening, nuchal translucency was 1.49 mm. Ultrasound examination revealed IUGR and microcephaly at 34 + 3 weeks with an estimated fetal weight of 1,695 g (−2.7 SD) (Figure 1A) and a head circumference of 274 mm (−3.6 SD) (Figure 1B). The child was born at 39 + 2 weeks gestation by vaginal delivery. The birth weight was 2,460 g (−2.2 SD), and the Apgar score was 9 at 0 min and 9 at 5 min (the reference population was Asian and Pacific Islander from the National Institute of Child Health and Human Development Fetal Growth Study (Buck Louis et al., 2015)). At 2 months, she was transferred to the pediatric intensive care unit (ICU) with acute wheezing bronchitis, developmental delay, and seizure; her body weight was 2,440 g (−3 SD) and head circumference was 32 cm (−3 SD) (the reference population was Chinese children (Zong and Li, 2013)). The physical examination found craniosynostosis with an anterior fontanelle (0.5 × 0.5 cm) and hypertonia. Arthrogryposis of the index fingers and thumbs was observed. The results of routine tests such as liver function, blood glucose, renal function, and newborn screening by tandem mass spectrometry were normal, and she passed the auditory brainstem response test. An MRI revealed a thin corpus callosum (Figure 1C), hypomyelination (Figure 1D), and decreased craniofacial ratio. No other abnormalities were found. She passed away 1 week after ICU admission from respiratory failure.

## Molecular Findings

Peripheral blood samples were collected from the proband and her parents for trio-whole exome sequencing (WES). Genomic DNA was extracted from the blood samples using the SolPure Blood DNA Kit (Guangzhou Magen Biotechnology Co., Guangzhou, China). Exome capture was performed using xGen Exome Research panel v1 (Integrated DNA Technologies, Coralville, IA, United States), and sequencing was performed using a NovaSeq 6,000 system (Illumina, San Diego, CA, United States). The sequences were aligned to a human reference sequence (NCBI Genome build GRCh37) with the Burrows–Wheeler Aligner (0.7.10-r789) (Li and Durbin, 2010), and coverage above 20× was >98%. The Genome Analysis Toolkit (4.1.8) pipeline was used to detect single-nucleotide and insertion/deletion (INDEL) polymorphisms (McKenna et al., 2010), with 39,816 variants identified. The variants were annotated with ANNOVAR (2019–10-24) according to GRCh37 (Wang et al., 2010), and variant interpretation was performed according to the American College of Medical Genetics and Genomics/Association for Molecular Pathology guidelines and Clinical Genome Resource specifications (Richards et al., 2015; Zhang et al., 2020). We prioritized variants that were previously reported, considered loss-of-function (nonsense, frameshift, or splice sites mutations) or absent in gnomAD. IUGR (Human Phenotype Ontology [HP]: 0001511), central nervous system hypomyelination (HP: 0003429), seizure (HP: 0001250), and microcephaly (HP: 0000252) were used to narrow down the candidate gene list. We identified a novel homozygous nonsense variant in *SMPD4* (NM_017951.4: c.1347C > G [p.Tyr449*]). This variant had a CADD_PHRED score of 34 and was absent in gnomAD (PM2_Supporting) and was predicted to cause a premature termination in exon 14 of 20 that likely results in nonsense-mediated mRNA decay (PVS1). The variant was, therefore, classified as likely pathogenic. The 3-dimensional structure of the SMPD4 protein was obtained using the AlphaFold Protein Structure database (Varadi et al., 2022), and mutations were predicted using ChimeraX1.3 (Pettersen et al., 2021) (Supplementary Figure S1A). The mutation was heterozygous in the mother and absent in the father, suggesting that it was hemizygous in the setting of a paternal deletion. Sanger sequencing was performed to confirm the result (Figure 1E). NextGENe software (SoftGenetics, State College, PA, United States) was used to analyze WES copy number variations (CNVs), which revealed a paternally inherited 344-kb contiguous gross deletion (Chr2 [GRCh37]: g.130877574_131221737del) that encompassed the whole *SMPD4* gene (Figure 1F). This region contained part or all of 29 genes, two of which had Online MIM phenotypes (SMPD4 and coiled-coil domain–containing 115 [CCDC115]), with no haploinsufficient genes identified. CCDC115 is responsible for the congenital disorder of glycosylation, type IIo (MIM: 616828), an autosomal recessive metabolic disorder characterized by infantile onset of progressive liver failure, hypotonia, and delayed psychomotor development. There were no rare variants of the *CCDC115* gene. Consequently, the c.1347C > G mutation was determined to be hemizygous, and *SMPD4* was identified as the causative gene in our patient. The steps and methodology for molecular diagnosis are summarized in Supplementary Figure S1B.

## Discussion and Conclusion

*SMPD4* encodes sphingomyelin phosphodiesterase 4 (SMPD4), which preferentially hydrolyzes sphingomyelin over other phospholipids (Krut et al., 2006). Sphingomyelin is required for the proper functioning of the nervous system; an imbalance between sphingomyelin synthesis and degradation has been linked to a variety of neurologic pathologies including Niemann–Pick disease and Alzheimer’s disease (Bienias et al., 2016). In fibroblasts derived from affected individuals, *SMPD4* deletion results in aberrant mitosis and increased susceptibility to apoptotic cell death (Magini et al., 2019), which are mechanisms that have been shown to underlie human microcephaly and a simplified gyral pattern (Adachi et al., 2011).

To date, 20 *SMPD4* variants have been identified in 29 genetically confirmed individuals from 16 unrelated families (including our case) (Table 1); 28 individuals presented part or all of the manifestations including IUGR, microcephaly, arthrogryposis, a thin corpus callosum, and a simplified gyral pattern. However, only one individual showed distinct symptoms of brain atrophy and skeletal dysplasia (UPN-1246) (Monies et al., 2019). Notably, this individual shared the same homozygous null variant with another case of Arab descent (Family 5) who presented typical symptoms. Thus, skeletal dysplasia and brain atrophy are not variant-specific features. No animal models are currently available to confirm the phenotypes of *SPMD4* knockout; therefore, additional cases and functional analyses are needed to determine why the same null mutation resulted in two distinct phenotypes.

**TABLE 1:** *SMPD4* variants and affected families.

Family	Ethnicity/origin	Phenotype	Variant(s) NM_017951.4	Variant type	Zygosity	References
UPN-0877	Arab-Saudi Arabia	Bilateral clenched hands and talipes, IUGR, and partial absence of corpus callosum. Three similarly affected relatives in the family	c.1777–28_1783del (p.?)	Splice site loss	Hom	Monies et al. (2019)
UPN-1246	Arab-Saudi Arabia	Postnatal developmental delay, brain atrophy, and bone abnormalities	c.390_406del (p.Pro131LeufsTer2)	Frameshift	Hom	Monies et al. (2019)
Family 1	Turkish	IUGR, microcephaly, SGP, thin corpus callosum, hypomyelination, hypotonia, and early demise. Four affected relatives in the family	c.1407-9G > A (p.?)	Splice region (confirmed by RNA-sequencing)	Hom	Magini et al. (2019)
Family 2	Morocco	Microcephaly with SGP, delayed myelination, thin corpus callosum, brainstem and cerebellar hypoplasia, and severe intellectual disability	c.1570+1G > A (p.?)	Splice site mutation	Hom	Magini et al. (2019)
Family 3	USA/European	Microcephaly, seizure, vertical talus, and died in infancy	c.462+1G > T (p.?)	Splice site mutation	Het	Magini et al. (2019)
			c.199C > T (p.Gln67*)	Nonsense	Het	
Family 4	Arab-Saudi Arabia	IUGR, lissencephaly, cerebellar hypoplasia, hypotonia, contractures of fingers, and rocker bottom feet.	c.2281C > T (p.Gln761*)	Nonsense	Hom	Magini et al. (2019)
Family 5	Arab-Saudi Arabia	IUGR, microcephaly with moderate SGP, relatively small cerebellum, contractures of fingers, and rocker bottom feet.	c.390_406del (p.Pro131LeufsTer2)	Frameshift	Hom	Magini et al. (2019)
Family 6	Arab	IUGR, multiple joint contractures, and twins of dichorionic diamniotic pregnancy were affected. Early demise	c.244–2A > G (p.?)	Splice site mutation	Hom	Magini et al. (2019)
Family 7	European	IUGR, seizure, microcephaly, hypotelorism, arthrogryposis, adducted thumbs, and hypertrichosis of lower back. Early demise	c.692T > C (p.Leu231Pro)	Missense	Hemi	Magini et al. (2019)
			Chr2 [GRCh37]:g.129829959_131404737del	Whole-gene deletion	Het	
Family 8	Arab-Kuwait	Microcephaly with SGP and small cerebrum. Thin corpus callosum, delayed myelination, borderline small brainstem, hypertonicity, contractures, and seizures	c.370G > T (p.Glu124*)	Nonsense	Hom	Magini et al. (2019)
Family 9	Egyptian	IUGR, seizure, abnormal gyral pattern, thin corpus callosum, syndactyly, hypotonia, and mild autistic behavior. Two affected individuals, survived after the first decade	c.1337C > T (p.Pro446Leu)	Missense	Hom	Magini et al. (2019)
Family 10	Arab	SGA, microcephaly, dysmorphism, clenched hands, bilateral talipes, thin corpus callosum, and abnormal cerebellar folia	c.1473_1479del (p.Leu492Glnfs*27)	Frameshift	Hom	Magini et al. (2019)
Family 11	European	Bilateral contractures of fingers and toes, bilateral club feet, hypotonia, and can speak in sentences. Two affected individuals, alive at last follow-up	c.1982C > T (p.Ala661Val)	Missense	Het	Magini et al. (2019)
			c.387-8G > A (p.?)	Splice region	Het	
Family 12	Tunisian Jews	IUGR, polyhydramnios, bilateral clubfoot, and clenched hands. Two affected fetuses, TOP.	c.2088del (p.Asp697Thrfs*95)	Frameshift	Het	Magini et al. (2019)
			c.387-1G > C (p.?)	Splice site mutation	Het	
M-family	Australia	SGA, hypoplasia of the corpus callosum, arthrogryposis multiplex congenita, microcephaly, cerebellar malformation, and hypomyelination. Three affected individuals in the family. One early demise and two TOP.	c.575C > T (p.Pro192Leu)	Missense	Hom	Ravenscroft et al. (2020)
C-family	China	IUGR, microcephaly, hypomyelination, hypertonicity, seizure, and early demise	c.1347C > G (p.Tyr449*)	Nonsense	Hemi	This study
			Chr2 [GRCh37]: g.130877574_131221737del	Whole-gene deletion	Het	

Abbreviations: Hemi, hemizygous; Het, heterozygous; Hom, homozygous; IUGR, intrauterine growth restriction; SGP, simplified gyral pattern; SGA, small for gestational age; TOP, termination of pregnancy.

All reported *SMPD4* variants are summarized in Table 1; Figure 1F including nonsense mutations (4/20), splice site/region mutations (7/20), frameshifts (3/20), missense mutations (4/20), and gross deletions (2/20); of these, 15/20 are null mutations. Individuals with biallelic null mutations always exhibit more severe phenotypes, such as brain structural abnormalities, arthrogryposis, and early death (Magini et al., 2019; Monies et al., 2019; Ravenscroft et al., 2020). Four individuals from two families (Families 9 and 11) harboring non-null mutations survived into childhood and showed some motor skill and mental development (Magini et al., 2019). Our proband carried the compound heterozygous nonsense mutation and a whole-gene deletion, both of which abolished the protein and appeared to cause severe neonatal developmental delay, microcephaly with craniosynostosis, and early demise, which are among the most severe manifestations of NEDMABA.

Although only 28 individuals with NEDMABA have been reported, this may be an underestimate; the prevalence estimated based on the gene carrier rate (Guo and Gregg, 2019) calculated from loss-of-function variants of *SMPD4* in gnomAD is about one in 1,580,000. This may be attributable to the fact that the typical symptoms of NEDMABA are nonspecific, making clinical diagnosis difficult. In many countries, chromosomal microarray is the first-tier genetic test for individuals with developmental disabilities or congenital anomalies, with diagnosis rates of 10–20% (Miller et al., 2010). Since 2011, WES has been increasingly used to determine the etiology of genetic disorders; more than 20% of patients can be diagnosed using this method (Yang et al., 2013; Meng et al., 2017). WES can identify single-nucleotide variations (SNVs) and small INDELs. However, large CNVs are missed by the standard analysis pipeline. Recently, several algorithms for WES-based CNV detection have been developed based on comparisons of depth of coverage (Yang et al., 2013; Backenroth et al., 2014; Fromer and Purcell, 2014; Talevich et al., 2016; Meng et al., 2017) and could detect the CNVs of the exon level which are smaller than those detected by CMA. Thus, WES could replace chromosomal microarray as a more cost-effective genetic test for detecting CNVs and diagnosing highly heterogenous conditions such as NEDMABA. In our case, the standard pipeline only identified a maternally inherited homozygous variant. There are several possible explanations for this observation including uniparental disomy, *de novo* mutation, and deletion of the corresponding region on another allele. The risk to the siblings of the affected individual should be discussed in genetic counseling sessions, although that is likely to vary according to the situation. Based on the paternally inherited deletion identified by CNV WES, we predict a 25% risk.

In conclusion, our study reveals for the first time the NEDMABA phenotype of an individual of Chinese ancestry and provides further evidence for the role of *SMPD4* in this syndrome.

## Data Availability

The datasets for this article are not publicly available due to concerns regarding participant/patient anonymity. Requests to access the datasets should be directed to the corresponding author.

## References

[B1] Adachi Y. Poduri A. Kawaguch A. Yoon G. Salih M. A. Yamashita F. (2011). Congenital Microcephaly with a Simplified Gyral Pattern: Associated Findings and Their Significance. AJNR Am. J. Neuroradiol. 32, 1123–1129. 10.3174/ajnr.A2440 21454410 PMC3838394

[B2] Backenroth D. Homsy J. Murillo L. R. Glessner J. Lin E. Brueckner M. (2014). CANOES: Detecting Rare Copy Number Variants from Whole Exome Sequencing Data. Nucleic Acids Res. 42, e97. 10.1093/nar/gku345 24771342 PMC4081054

[B3] Bienias K. Fiedorowicz A. Sadowska A. Prokopiuk S. Car H. (2016). Regulation of Sphingomyelin Metabolism. Pharmacol. Rep. 68, 570–581. 10.1016/j.pharep.2015.12.008 26940196

[B4] Buck Louis G. M. Grewal J. Albert P. S. Sciscione A. Wing D. A. Grobman W. A. (2015). Racial/Ethnic Standards for Fetal Growth: The NICHD Fetal Growth Studies. Am. J. Obstet. Gynecol. 213, 449.e1–449.e41. 10.1016/j.ajog.2015.08.032 26410205 PMC4584427

[B5] Fromer M. Purcell S. M. (2014). Using XHMM Software to Detect Copy Number Variation in Whole‐Exome Sequencing Data. Curr. Protoc. Hum. Genet. 81, 7.23.1–7.23.21. 10.1002/0471142905.hg0723s81 24763994 PMC4065038

[B6] Guo M. H. Gregg A. R. (2019). Estimating Yields of Prenatal Carrier Screening and Implications for Design of Expanded Carrier Screening Panels. Genet. Med. 21, 1940–1947. 10.1038/s41436-019-0472-7 30846881

[B7] Krut O. Wiegmann K. Kashkar H. Yazdanpanah B. Krönke M. (2006). Novel Tumor Necrosis Factor-Responsive Mammalian Neutral Sphingomyelinase-3 Is a C-Tail-Anchored Protein. J. Biol. Chem. 281, 13784–13793. 10.1074/jbc.M511306200 16517606

[B8] Li H. Durbin R. (2010). Fast and Accurate Long-Read Alignment with Burrows-Wheeler Transform. Bioinformatics 26, 589–595. 10.1093/bioinformatics/btp698 20080505 PMC2828108

[B9] Magini P. Smits D. J. Vandervore L. Schot R. Columbaro M. Kasteleijn E. (2019). Loss of SMPD4 Causes a Developmental Disorder Characterized by Microcephaly and Congenital Arthrogryposis. Am. J. Hum. Genet. 105, 689–705. 10.1016/j.ajhg.2019.08.006 31495489 PMC6817560

[B10] McKenna A. Hanna M. Banks E. Sivachenko A. Cibulskis K. Kernytsky A. (2010). The Genome Analysis Toolkit: A MapReduce Framework for Analyzing Next-Generation DNA Sequencing Data. Genome Res. 20, 1297–1303. 10.1101/gr.107524.110 20644199 PMC2928508

[B11] Meng L. Pammi M. Saronwala A. Magoulas P. Ghazi A. R. Vetrini F. (2017). Use of Exome Sequencing for Infants in Intensive Care Units. JAMA Pediatr. 171, e173438. 10.1001/jamapediatrics.2017.3438 28973083 PMC6359927

[B12] Miller D. T. Adam M. P. Aradhya S. Biesecker L. G. Brothman A. R. Carter N. P. (2010). Consensus Statement: Chromosomal Microarray Is a First-Tier Clinical Diagnostic Test for Individuals with Developmental Disabilities or Congenital Anomalies. Am. J. Hum. Genet. 86, 749–764. 10.1016/j.ajhg.2010.04.006 20466091 PMC2869000

[B13] Monies D. Abouelhoda M. Assoum M. Moghrabi N. Rafiullah R. Almontashiri N. (2019). Lessons Learned from Large-Scale, First-Tier Clinical Exome Sequencing in a Highly Consanguineous Population. Am. J. Hum. Genet. 105, 879. 10.1016/j.ajhg.2019.04.01110.1016/j.ajhg.2019.09.019 31585110 PMC6817532

[B14] Pettersen E. F. Goddard T. D. Huang C. C. Meng E. C. Couch G. S. Croll T. I. (2021). UCSF ChimeraX : Structure Visualization for Researchers, Educators, and Developers. Protein Sci. 30, 70–82. 10.1002/pro.3943 32881101 PMC7737788

[B15] Ravenscroft G. Clayton J. S. Faiz F. Sivadorai P. Milnes D. Cincotta R. (2020). Neurogenetic Fetal Akinesia and Arthrogryposis: Genetics, Expanding Genotype-Phenotypes and Functional Genomics. J. Med. Genet. 58, 609–618. 10.1136/jmedgenet-2020-106901 33060286 PMC8328565

[B16] Richards S. Aziz N. Bale S. Bick D. Das S. Gastier-Foster J. (2015). Standards and Guidelines for the Interpretation of Sequence Variants: A Joint Consensus Recommendation of the American College of Medical Genetics and Genomics and the Association for Molecular Pathology. Genet. Med. 17, 405–424. 10.1038/gim.2015.30 25741868 PMC4544753

[B17] Talevich E. Shain A. H. Botton T. Bastian B. C. (2016). CNVkit: Genome-Wide Copy Number Detection and Visualization from Targeted DNA Sequencing. Plos Comput. Biol. 12, e1004873. 10.1371/journal.pcbi.1004873 27100738 PMC4839673

[B18] Varadi M. Anyango S. Deshpande M. Nair S. Natassia C. Yordanova G. (2022). AlphaFold Protein Structure Database: Massively Expanding the Structural Coverage of Protein-Sequence Space with High-Accuracy Models. Nucleic Acids Res. 50, D439–D444. 10.1093/nar/gkab1061 34791371 PMC8728224

[B19] Wang K. Li M. Hakonarson H. (2010). ANNOVAR: Functional Annotation of Genetic Variants from High-Throughput Sequencing Data. Nucleic Acids Res. 38, e164. 10.1093/nar/gkq603 20601685 PMC2938201

[B20] Yang Y. Muzny D. M. Reid J. G. Bainbridge M. N. Willis A. Ward P. A. (2013). Clinical Whole-Exome Sequencing for the Diagnosis of Mendelian Disorders. N. Engl. J. Med. 369, 1502–1511. 10.1056/NEJMoa1306555 24088041 PMC4211433

[B21] Zhang J. Yao Y. He H. Shen J. (2020). Clinical Interpretation of Sequence Variants. Curr. Protoc. Hum. Genet. 106, e98. 10.1002/cphg.98 32176464 PMC7431429

[B22] Zong X.-N. Li H. (2013). Construction of a New Growth References for China Based on Urban Chinese Children: Comparison with the WHO Growth Standards. PLoS One 8, e59569. 10.1371/journal.pone.0059569 23527219 PMC3602372

