# Supplementary figures and images for: Case Report: Novel Biallelic Null Variants of SMPD4 Confirm Its Involvement in Neurodevelopmental Disorder With Microcephaly, Arthrogryposis, and Structural Brain Anomalies
Source: Front Genet. 2022 May 16;13:872264. doi: 10.3389/fgene.2022.872264 (PMC9149365; doi:10.3389/fgene.2022.872264)

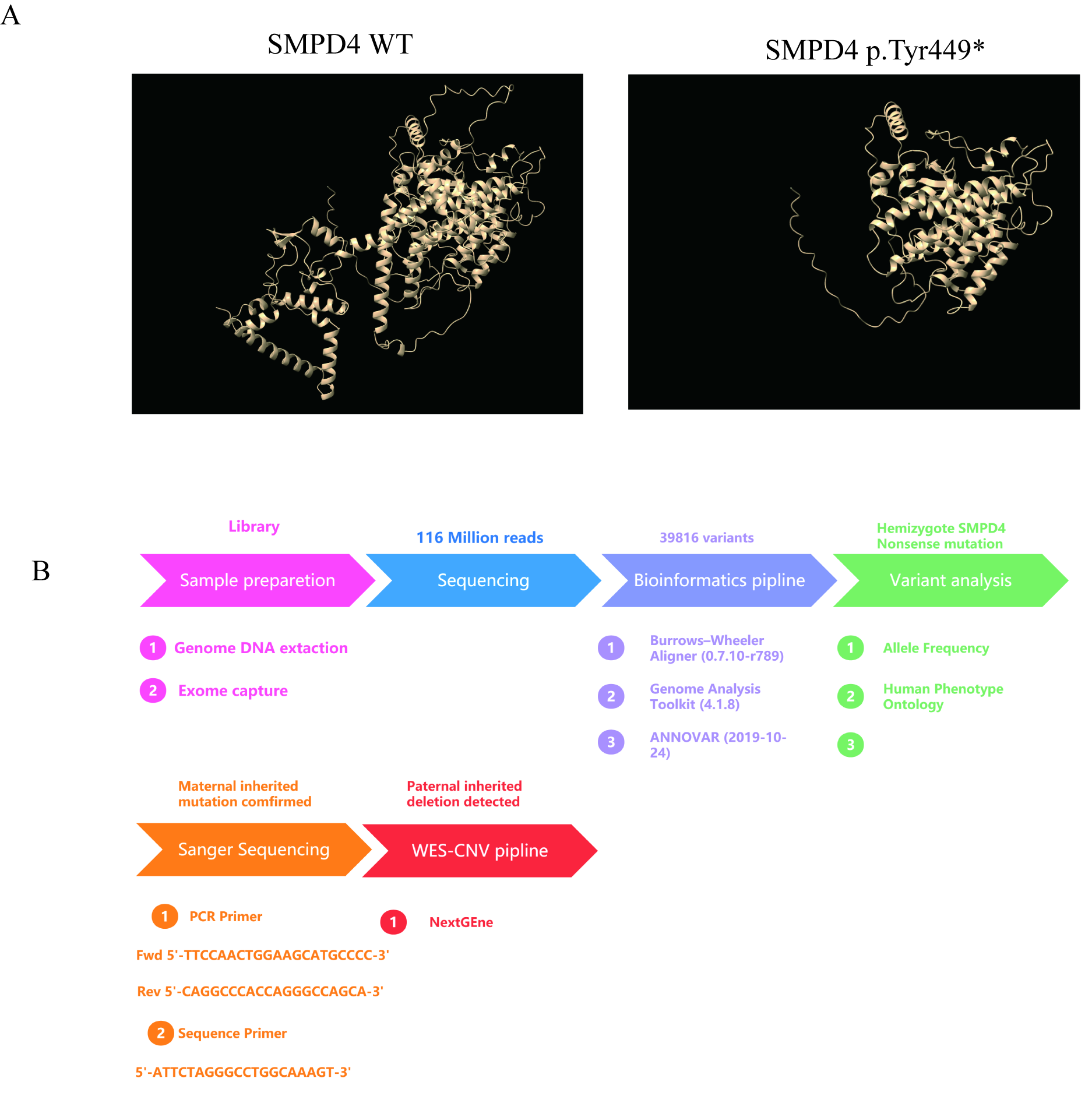

Supplement: Supplementary file 1 [file Image1.TIF]
